# Supplementary material for: α/β-Hydrolase domain-containing 6 (ABHD6) accelerates the desensitization and deactivation of TARP γ-2-containing AMPA receptors
Source: eLife. 2026 Aug 18;13:RP99623. doi: 10.7554/eLife.99623 (PMC13485305; doi:10.7554/eLife.99623)
Supplement: Supplementary file 2 [file elife-99623-supp1.docx]

Supplementary File 1. Summary of peak amplitude (pA) of GluAs when co-transfected with/without γ-2 or/and ABHD6.

| **Subunit** | **GluA** | | **GluA**  **+ ABHD6** | | **GluA**  **+ TARP γ-2** | | **GluA**  **+ TARP γ-2**  **+ ABHD6** | |
| --- | --- | --- | --- | --- | --- | --- | --- | --- |
| GluA1i | 226.69 ± 167.55(32/4) | | 77.91 ± 76.58(36/4)^a^ | | 1005.89 ± 530.81(33/4)^a^ | | 135.92 ± 140.83(36/4)^b^ | |
| GluA1o | 88.67 ± 59.73(33/4) | | 42.62 ± 48.11(38/4)^a^ | | 309.24 ± 211.47(37/4)^a^ | | 115.51 ± 126.12(30/4)^b^ | |
| GluA2(Q)i-R | 281.51 ± 256.66(36/5) | | 87.50 ± 81.75(42/5)^a^ | | 911.62 ± 607.39(38/5)^a^ | | 77.86 ± 157.14(44/5)^b^ | |
| GluA2(Q)o-R | 201.23 ± 171.09(38/5) | | 63.84 ± 95.32(46/5)^a^ | | 736.02 ± 579.63(34/5)^a^ | | 82.15 ± 205.29(41/5)^b^ | |
| GluA2(Q)i-G | 211.76 ± 254.32(38/5) | | 95.45 ± 119.83(38/5)^a^ | | 660.77 ± 389.82(38/5)^a^ | | 42.89 ± 59.48(43/5)^b^ | |
| GluA2(Q)o-G | 196.15 ± 175.99(48/6) | | 41.57 ± 39.21(45/6)^a^ | | 567.72 ± 361.19(46/6)^a^ | | 37.75 ± 56.79(49/6)^b^ | |
| GluA2(R)i-R | 2.55 ± 3.32(26/4) | | 1.86 ± 1.27(30/3) | | 55.83 ± 52.57(39/4)^a^ | | 5.34 ± 6.69(30/3)^b^ | |
| GluA2(R)o-R | 1.72 ± 1.51(14/3) | | 1.72 ± 0.88(29/3) | | 28.69 ± 32.66(34/4)^a^ | | 12.63 ± 19.49(26/4)^b^ | |
| GluA2(R)i-G | 2.60 ± 3.59(33/4) | | 2.55 ± 2.56(33/3) | | 31.32 ± 35.76(36/4)^a^ | | 3.29 ± 4.82(33/3)^b^ | |
| GluA2(R)o-G | 2.57 ± 2.41(43/5) | | 1.95 ± 1.62(49/4) | | 9.26 ± 9.75(48/5)^a^ | | 1.95 ± 1.52(49/4)^b^ | |
| GluA3i-R | 21.11 ± 40.80(26/3) | | 3.29 ± 5.57(20/2) | | 24.28 ± 46.24(25/3) | | 1.22 ± 1.15(21/2)b | |
| GluA3o-R | 6.10 ± 5.89(25/3) | | 2.46 ± 2.27(20/2) | | 11.38 ± 15.56(25/3) | | 4.36 ± 11.04(21/2)^b^ | |
| GluA3i-G | 9.83 ± 16.73(25/3) | | 3.32 ± 4.01(19/2) | | 15.27 ± 17.49(26/3)^a^ | | 3.25 ± 2.83(19/2)^b^ | |
| GluA3o-G | 3.12 ± 4.66(25/3) | | 1.15 ± 0.67(20/2) | | 4.70 ± 7.75(26/3) | | 1.15 ± 0.95(20/2)^b^ | |
| GluA2(Q)i-R-TARP γ-2 | | 1222.38 ± 96.94(73/9) | | 393.40 ± 376.66 (73/9) | | \ | | \ |
| GluA1i-TARP γ-2 | | 1073.10 ± 153.50(36/3) | | 267.62 ± 54.62 (36/3) | | \ | | \ |
| GluA2(Q)i-R-TARP γ-2  (+ 50 mV) | | 315.01 ± 243.25(74/9) | | 142.63 ± 130.52(71/9) | | \ | | \ |
| GluA1i-TARP γ-2  (+ 50 mV) | | 181.08 ± 142.67(36/3) | | 60.06 ± 55.86(36/3) | | \ | | \ |

Mean (± S.D.) values for the peak amplitude of glutamate-induced currents, which were calculated from the desensitization curves, number of cells, and batches, are in brackets. And the curves from a double exponential function fit to the glutamate-induced currents evoked by 100 ms applications of 10 mM glutamate. Currents were recorded in outside-out patch recordings (-60 mV) from cells transfected with GluA, GluA + ABHD6, GluA + TARP γ-2, and GluA + TARP γ-2 + ABHD6. The number of patches is given in parentheses. Data were analyzed for normality by Shapiro-Wilk test (n＜50) or Kolmogorov-Smirnov test (n＞50) and Brown-Forsythe ANOVA test reported equality of variances: GluA1i(F=(3.000, 45.28)73.46, P＜0.0001), GluA1o(F=30.76 (3.000, 69.40), P＜0.0001), GluA2(Q)i-R(F=32.96 (3.000, 84.61), P＜0.0001), GluA2(Q)o-R(F=37.04 (3.000, 58.80), P＜0.0001), GluA2(Q)i-G(F=47.66 (3.000, 81.39), P＜0.0001), GluA2(Q)o-G(F=28.48 (3.000, 121.6), P＜0.0001), GluA2(R)i-R(F=38.19 (3.000, 39.76), P＜0.0001), GluA2(R)o-R(F=13.82 (3.000, 53.60), P＜0.0001), GluA2(R)i-G(F=22.12 (3.000, 37.43), P＜0.0001), GluA2(R)o-G(F=23.11 (3.000, 58.11), P＜0.0001), GluA3i-R(F=3.480 (3.000, 48.76), P=0.0228), GluA3o-R(F=3.415 (3.000, 50.87), P=0.0241), GluA3i-G(F= 5.191 (3.000, 53.36), P= 0.0032), GluA3o-G(F= 3.421 (3.000, 42.46), P= 0.0256), GluA2(Q)i-R-TARP γ-2(F= 4.836, 72, 72, P＜0.0001), GluA1i-TARP γ-2(F= 3.473, 73, 70, P＜0.0001), TARP γ-2(+ 50 mV) (F= 7.898, 35, 35, P＜0.0001) and GluA1i-TARP γ-2(+ 50 mV) (F= 6.522, 35, 35, P＜0.0001).

^a^Significantly different from GluA.

^b^Significantly different from GluA + TARP γ-2.
