# Supplementary material for: α/β-Hydrolase domain-containing 6 (ABHD6) accelerates the desensitization and deactivation of TARP γ-2-containing AMPA receptors
Source: eLife. 2026 Aug 18;13:RP99623. doi: 10.7554/eLife.99623 (PMC13485305; doi:10.7554/eLife.99623)
Supplement: Supplementary file 4 [file elife-99623-supp11.docx]

Supplementary File 11 Summary of GluA2(R)i-G/GluA3(R)i receptors when co-transfected with/without γ-2 or/and ABHD6.

|  | **GluA2(R)i-G/GluA3(R)i** | **GluA2(R)i-G/GluA3(R)i + ABHD6** | **GluA2(R)i-G/GluA3(R)i + TARP γ-2** | **GluA2(R)i-G/GluA3(R)i + TARP γ-2 + ABHD6** |
| --- | --- | --- | --- | --- |
| Peak amplitude | 132.3 ±55.74 (19/3) | 42.11 ±34.19 (18/3) ^a^ | 514.2 ±171.1 (20/3)^a^ | 66.42 ±67.24 (18/3)^b^ |
| *τ* _w,_ _deact_ | 1.128 ±0.2640 (19/3) | 1.159 ±0.3888 (18/3) | 2.325 ±0.8924 (20/3)^a^ | 1.284 ±0.3922 (18/3)^b^ |
| τ _w, des_ | 5.346 ±1.203 (19/3) | 4.949 ±2.735 (18/3) | 7.645 ±1.579 (20/3)^a^ | 5.160 ±3.823 (18/3)^b^ |
| τ _rec_ | 75.46 ±89.31 (18/3) | 57.58 ±23.78 (18/3)^a^ | 81.31 ±35.58 (19/3)^a^ | 89.81 ±49.89 (18/3)^b^ |

Mean (± S.D.) values for peak amplitude, τ _w, des_ from the desensitization and *τ* _w,_ _deact_ from the deactivation curves, and τ _rec_ from the recovery ratio curves. Currents were recorded in outside-out patch recordings (- 60 mV, 10 mM glutamate; 100 ms / 1 ms / 100 two-pulse stimulation) without spermine in internal solution, from cells transfected with GluA2(R)i-G/GluA3(R)i, GluA2(R)i-G/GluA3(R)i + ABHD6, GluA2(R)i-G/ GluA3(R)i + TARP γ-2, and GluA2(R)i-G/GluA3(R)i + TARP γ-2 + ABHD6. The number of patches is given in parentheses.

^a^Significantly different from GluA2(R)i-G/ GluA3(R)i.

^b^Significantly different from GluA2(R)i-G/ GluA3(R)i + TARP γ-2.
