# Supplementary material for: α/β-Hydrolase domain-containing 6 (ABHD6) accelerates the desensitization and deactivation of TARP γ-2-containing AMPA receptors
Source: eLife. 2026 Aug 18;13:RP99623. doi: 10.7554/eLife.99623 (PMC13485305; doi:10.7554/eLife.99623)
Supplement: Supplementary file 6 [file elife-99623-supp13.docx]

Supplementary File 13 Summary of wild-type primary hippocampal neurons and ABHD6 knockout primary hippocampal neurons.

|  | **WT** | **KO** |
| --- | --- | --- |
| Peak amplitude | 243.2 ±264.5 (43/3) | 569.9±650.5 (44/3) |
| *τ* _w,_ _deact_ | 2.163 ±0.8521 (43/3) | 2.616 ±1.044 (44/3) |
| τ _w, des_ | 4.406 ±2.284 (43/3) | 6.514 ±4.646 (44/3) |
| τ _rec_ | 133.3 ±122.0 (38/3) | 89.87 ±54.54 (43/3) |

Mean (± S.D.) values for peak amplitude, τ _w, des_ from the desensitization and *τ* _w,_ _deact_ from the deactivation curves, and τ _rec_ from the recovery ratio curves. Currents were recorded in outside-out patch recordings (- 70 mV, 10 mM glutamate; 100 ms / 1 ms / 100 two-pulse stimulation) without spermine in internal solution from cultured WT or ABHD6 KO primary hippocampal neurons. The number of patches is given in parentheses.
