# Supplementary material for: α/β-Hydrolase domain-containing 6 (ABHD6) accelerates the desensitization and deactivation of TARP γ-2-containing AMPA receptors
Source: eLife. 2026 Aug 18;13:RP99623. doi: 10.7554/eLife.99623 (PMC13485305; doi:10.7554/eLife.99623)
Supplement: Supplementary file 7 [file elife-99623-supp14.docx]

Supplementary File 14 Summary of P values for comparison of wild-type primary hippocampal neuron and ABHD6 knockout primary hippocampal neuron.

|  | **WT vs. KO** |
| --- | --- |
| Peak amplitude | P=0.0012 (**) |
| *τ* _w,_ _deact_ | P=0.0434 (*) |
| τ _w, des_ | P=0.0456 (*) |
| τ _rec_ | P=0.0526(-) |

P values for comparison of peak amplitude, *τ* _w,_ _deact_, τ _w, des_, and τ _rec_ of WT or ABHD6 KO primary hippocampal neurons. The data were analyzed using Prism 8 (GraphPad Software), and each P value was adjusted to account for multiple comparisons.
