# Supplementary material for: α/β-Hydrolase domain-containing 6 (ABHD6) accelerates the desensitization and deactivation of TARP γ-2-containing AMPA receptors
Source: eLife. 2026 Aug 18;13:RP99623. doi: 10.7554/eLife.99623 (PMC13485305; doi:10.7554/eLife.99623)
Supplement: Supplementary file 8 [file elife-99623-supp15.docx]

Supplementary File 15 Summary of GluA4i receptors when co-transfected with/without γ-2 or/and ABHD6.

|  | **GluA4i** | **GluA4i + ABHD6** | **GluA4i + TARP γ-2** | **GluA4i + TARP γ-2 + ABHD6** |
| --- | --- | --- | --- | --- |
| Peak amplitude | 443.2 ±414.8 (39/3) | 77.40 ±133.6 (37/3) ^a^ | 607.3 ±464.9 (29/3)^a^ | 101.1 ±125.3 (37/3)^b^ |
| *τ* _w,_ _deact_ | 1.411 ±1.006 (40/3) | 0.9040 ±0.5214 (36/3) ^a^ | 2.879 ±2.420 (39/3) ^a^ | 2.274 ±1.805 (37/3)^b^ |
| τ _w, des_ | 3.533 ±0.6888 (40/3) | 2.763 ±1.229 (36/3) | 7.075 ±1.951 (39/3)^a^ | 4.907 ±2.313 (35/3)^b^ |
| τ _rec_ | 44.55 ±15.95 (36/3) | 55.44 ±25.19 (31/3)^a^ | 56.24 ±39.57 (36/3)^a^ | 62.53 ±41.96 (29/3)^b^ |

Mean (± S.D.) values for peak amplitude, τ _w, des_ from the desensitization and *τ* _w,_ _deact_, from the deactivation curves, and τ _rec_ from the recovery ratio curves. Currents were recorded in outside-out patch recordings (- 60 mV, 10 mM glutamate; 100 ms / 1 ms / 100 two-pulse stimulation) without spermine in internal solution, from cells transfected with GluA4i, GluA4i + ABHD6, GluA4i + TARP γ-2, GluA4i + TARP γ-2 + ABHD6. The number of patches is given in parenthesis.

^a^Significantly different from GluA4i.

^b^Significantly different from GluA4i + TARP γ-2.
