# Supplementary material for: α/β-Hydrolase domain-containing 6 (ABHD6) accelerates the desensitization and deactivation of TARP γ-2-containing AMPA receptors
Source: eLife. 2026 Aug 18;13:RP99623. doi: 10.7554/eLife.99623 (PMC13485305; doi:10.7554/eLife.99623)
Supplement: Supplementary file 9 [file elife-99623-supp16.docx]

Supplementary File 16 Summary of P values for comparison of GluA4i receptors when co-transfected with/without γ-2 or/and ABHD6.

|  | **GluA4i +ABHD6**  **vs. GluA4i** | **GluA4i +TARP γ-2 vs. GluA4i** | **GluA4i +TARP γ-2+ABHD6**  **vs. GluA4i +TARP γ-2** |
| --- | --- | --- | --- |
| Peak amplitude | P<0.001(***) | P=0.5472(-) | P>0.9999(-) |
| *τ* _w,_ _deact_ | P=0.2095(-) | P<0.001(***) | P=0.6753(-) |
| τ _w, des_ | P=0.1350(-) | P<0.001(***) | P=0.0006 (***) |
| τ _rec_ | P=0.1599(-) | P=>0.9999(-) | P=>0.9999(-) |

P values for comparison of Peak amplitude, *τ* _w,_ _deact_, τ _w, des_, τ _rec_ of GluA4i when co-transfected with/without γ-2 or/and ABHD6. The data were analyzed using Prism 8 (GraphPad Software), and each p values was adjusted to account for multiple comparisons.
