# Supplementary material for: α/β-Hydrolase domain-containing 6 (ABHD6) accelerates the desensitization and deactivation of TARP γ-2-containing AMPA receptors
Source: eLife. 2026 Aug 18;13:RP99623. doi: 10.7554/eLife.99623 (PMC13485305; doi:10.7554/eLife.99623)
Supplement: Supplementary file 10 [file elife-99623-supp17.docx]

Supplementary File 17 Summary of GluA1i receptors when co-transfected with/without γ-8 or/and ABHD6.

|  | **GluA1i** | **GluA1i + ABHD6** | **GluA1i + TARP γ-8** | **GluA1i + TARP γ-8 + ABHD6** |
| --- | --- | --- | --- | --- |
| Peak amplitude(nA) | 0.2909 ±0.2372 (45/3) | 0.1275 ±0.1793 (48/3) ^a^ | 0.8908 ±0.4672 (42/3)^a^ | 0.1330 ±0.1614 (46/3)^b^ |
| *τ* _w,_ _deact_ | 1.089 ±0.3320 (45/3) | 1.098 ±0.4644 (48/3) ^a^ | 3.577 ±1.483 (42/3) ^a^ | 2.319 ±1.140 (46/3)^b^ |
| τ _w, des_ | 3.209 ±1.019 (45/3) | 2.869 ±0.9605 (48/3) | 6.737 ±2.325 (42/3)^a^ | 4.620 ±1.780 (46/3)^b^ |
| τ _rec_ | 181.4 ±140.0 (40/3) | 185.8 ±84.66 (36/3)^a^ | 77.32 ±39.75 (41/3)^a^ | 78.35 ±53.36 (37/3)^b^ |

Mean (± S.D.) values for peak amplitude, τ _w, des_ from the desensitization and *τ* _w,_ _deact_, from the deactivation curves, and τ _rec_ from the recovery ratio curves. Currents were recorded in outside-out patch recordings (- 60 mV, 10 mM glutamate; 100 ms / 1 ms / 100 two-pulse stimulation) without spermine in internal solution, from cells transfected with GluA1i, GluA1i + ABHD6, GluA1i + TARP γ-8, GluA1i + TARP γ-8 + ABHD6. The number of patches is given in parenthesis.

^a^Significantly different from GluA1i.

^b^Significantly different from GluA4i + TARP γ-8.
