# Supplementary material for: α/β-Hydrolase domain-containing 6 (ABHD6) accelerates the desensitization and deactivation of TARP γ-2-containing AMPA receptors
Source: eLife. 2026 Aug 18;13:RP99623. doi: 10.7554/eLife.99623 (PMC13485305; doi:10.7554/eLife.99623)
Supplement: Supplementary file 12 [file elife-99623-supp19.docx]

Kunming Cell Bank, Chinese Academy of Sciences

Mycoplasma Test Report

Cell Line Name: 293T

Accession Number: KCB 200744YJ

Sample Type: Cells

Result: Fluorescent staining, mycoplasma negative

Image Results

20X	20X

Bright Field Image Results

10X	10X
