# Supplementary material for: α/β-Hydrolase domain-containing 6 (ABHD6) accelerates the desensitization and deactivation of TARP γ-2-containing AMPA receptors
Source: eLife. 2026 Aug 18;13:RP99623. doi: 10.7554/eLife.99623 (PMC13485305; doi:10.7554/eLife.99623)
Supplement: Supplementary file 13 [file elife-99623-supp2.docx]

Supplementary File 2 Summary of P values for comparison of peak amplitude.

| **Subunit** | **GluA+ABHD6**  **vs. GluA** | | **GluA+TARP γ-2 vs. GluA** | | **GluA+TARP γ-2+ABHD6**  **vs. GluA+TARP γ-2** | |
| --- | --- | --- | --- | --- | --- | --- |
| GluA1i | P=0.0037(**) | | P<0.0001(***) | | P<0.0001(***) | |
| GluA1o | P=0.0143(*) | | P<0.0001(***) | | P<0.0001(***) | |
| GluA2(Q)i-R | P=0.0045(**) | | P=0.0009(***) | | P<0.0001(***) | |
| GluA2(Q)o-R | P=0.0001(***) | | P=0.0018(**) | | P<0.0001(***) | |
| GluA2(Q)i-G | P=0.0070(**) | | P=0.0003(***) | | P<0.0001(***) | |
| GluA2(Q)o-G | P<0.0001(***) | | P=0.0008(***) | | P<0.0001(***) | |
| GluA2(R)i-R | P>0.9999(-) | | P<0.0001(***) | | P<0.0001(***) | |
| GluA2(R)o-R | P>0.9999(-) | | P<0.0001(***) | | P=0.0485(*) | |
| GluA2(R)i-G | P>0.9999(-) | | P<0.0001(***) | | P<0.0001(***) | |
| GluA2(R)o-G | P=0.1356(-) | | P<0.0001(***) | | P<0.0001(***) | |
| GluA3i-R | P=0.0007(-) | | P>0.9999(-) | | P<0.0001(***) | |
| GluA3o-R | P=0.0775(-) | | P>0.9999(-) | | P=0.0028(**) | |
| GluA3i-G | P>0.9999(-) | | P=0.0066(**) | | P=0.0005(***) | |
| GluA3o-G | P>0.9999(-) | | P=0.0838(-) | | P=0.0002(***) | |
| GluA2(Q)i-R-TARP γ-2 | | P<0.001(***) | | \ | | \ |
| GluA1i-TARP γ-2 | | P<0.001(***) | | \ | | \ |
| GluA2(Q)i-R-TARP γ-2  (+ 50 mV) | | P<0.001(***) | | \ | | \ |
| GluA1i-TARP γ-2  (+ 50 mV) | | P<0.001(***) | | \ | | \ |

P values for comparison of peak amplitude of GluAs when co-transfected with/without γ-2 or/and ABHD6. The data were analyzed using Prism 8 (GraphPad Software) by the Kruskal-Wallis test, and each P value was adjusted to account for multiple comparisons.
