# Supplementary material for: α/β-Hydrolase domain-containing 6 (ABHD6) accelerates the desensitization and deactivation of TARP γ-2-containing AMPA receptors
Source: eLife. 2026 Aug 18;13:RP99623. doi: 10.7554/eLife.99623 (PMC13485305; doi:10.7554/eLife.99623)
Supplement: Supplementary file 14 [file elife-99623-supp3.docx]

Supplementary File 3 Summary of *τ* _w,_ _deact_ (ms) of GluAs when co-transfected with/without γ-2 or/and ABHD6.

| **Subunit** | **GluA** | | **GluA**  **+ ABHD6** | | **GluA**  **+ TARP γ-2** | | **GluA**  **+ TARP γ-2**  **+ ABHD6** | |  |
| --- | --- | --- | --- | --- | --- | --- | --- | --- | --- |
| GluA1i | 0.99 ± 0.21(32/4) | | 0.98 ± 0.41(36/4) | | 2.88 ± 1.16(33/4)^a^ | | 1.38 ±0.38(36/4)^b^ | |  |
| GluA1o | 1.09 ± 0.27(33/4) | | 1.00 ± 0.35(36/4) | | 1.91 ± 0.81(37/4)^a^ | | 1.25 ± 0.49(30/4)^b^ | |  |
| GluA2(Q)i-R | 1.03 ± 1.05(36/5) | | 1.15 ±0.77(43/5) | | 3.68 ± 3.13(38/5)^a^ | | 1.78 ± 1.39(43/5)^b^ | |  |
| GluA2(Q)o-R | 0.97 ± 0.40(38/5) | | 1.07 ± 0.42(47/5) | | 2.78 ± 1.43(34/5)^a^ | | 1.66 ± 0.95(46/5)^b^ | |  |
| GluA2(Q)i-G | 0.96 ± 0.55(37/5) | | 0.81 ± 0.37(38/5) | | 2.44 ± 1.46(38/5)^a^ | | 1.79 ± 1.26(44/5) | |  |
| GluA2(Q)o-G | 0.68 ± 0.21(48/6) | | 0.76 ± 0.27(47/6) | | 1.90 ± 1.30(46/6)^a^ | | 1.14 ± 0.60(50/6)^b^ | |  |
| GluA2(Q)i-R-TARP γ-2 | | 3.23± 1.15(73/9) | | 2.34± 1.27(73/9) | | \ | | \ | |
| GluA1i-TARP γ-2 | | 4.19± 1.69(36/3) | | 2.31± 1.11(36/3) | | \ | | \ | |
| GluA2(Q)i-R-TARP γ-2  (+ 50 mV) | | 2.81 ± 1.80(74/9) | | 1.71 ± 0.93(71/9) | | \ | | \ | |
| GluA1i-TARP γ-2  (+ 50 mV) | | 2.64 ± 1.23(36/3) | | 1.86 ± 1.05(36/3) | | \ | | \ | |

Mean (± S.D.) values for the time constant of deactivation (*τ* _w,_ _deact_). The deactivation curves from a double exponential function fit to the glutamate-induced currents evoked by 1 ms applications of 10 mM glutamate. Currents were recorded in outside-out patch recordings (-60 mV) from cells transfected with GluA, GluA + ABHD6, GluA + TARP γ-2, and GluA + TARP γ-2 + ABHD6. The number of patches is given in parentheses.

^a^Significantly different from GluA.

^b^Significantly different from GluA + TARP γ-2.
