# Supplementary material for: α/β-Hydrolase domain-containing 6 (ABHD6) accelerates the desensitization and deactivation of TARP γ-2-containing AMPA receptors
Source: eLife. 2026 Aug 18;13:RP99623. doi: 10.7554/eLife.99623 (PMC13485305; doi:10.7554/eLife.99623)
Supplement: Supplementary file 16 [file elife-99623-supp5.docx]

Supplementary File 5 Summary of τ _w, des_ (ms) of GluAs when co-transfected with/without γ-2 or/and ABHD6.

| **Subunit** | **GluA** | **GluA**  **+ ABHD6** | **GluA**  **+ TARP γ-2** | **GluA**  **+ TARP γ-2**  **+ ABHD6** |
| --- | --- | --- | --- | --- |
| GluA1i | 2.91 ± 0.53(32/4) | 2.59 ± 0.78(36/4) | 6.72 ± 1.86(33/4)^a^ | 4.14 ± 1.35(36/4)^b^ |
| GluA1o | 3.39 ± 0.70(33/4) | 2.90 ± 0.69(38/4) | 5.52 ± 1.59(37/4)^a^ | 3.50 ± 1.15(30/4)^b^ |
| GluA2(Q)i-R | 6.82 ± 2.19(36/5) | 6.26 ± 1.83(42/5) | 15.43 ± 4.87(38/5)^a^ | 9.13 ± 3.41(44/5)^b^ |
| GluA2(Q)o-R | 2.82 ± 0.81(38/5) | 2.66 ± 0.78(46/5) | 4.71 ± 1.48(34/5)^a^ | 3.08 ± 1.33(41/5)^b^ |
| GluA2(Q)i-G | 7.00 ± 2.11(38/5) | 6.25 ± 2.0(38/5) | 15.85 ± 3.95(38/5)^a^ | 9.52 ± 3.93(43/5)^b^ |
| GluA2(Q)o-G | 1.36 ± 0.58(48/6) | 1.36 ± 0.51(45/6) | 2.01 ± 0.60(46/6)^a^ | 1.43 ± 0.50(49/6)^b^ |
| GluA2(Q)i-R-TARP γ-2 | 17.47± 4.68(73/9) | 11.78± 3.20(73/9) | \ | \ |
| GluA1i-TARP γ-2 | 8.78± 2.76(36/3) | 5.80± 2.12(36/3) | \ | \ |
| GluA2(Q)i-R-TARP γ-2  (+ 50 mV) | 15.52± 4.48(74/9) | 10.31 ± 3.71(71/9) | \ | \ |
| GluA1i-TARP γ-2  (+ 50 mV) | 6.81± 2.01(36/3) | 5.64 ± 1.85(36/3) | \ | \ |

Mean (± S.D.) values for the time constant of desensitization (τ _w, des_) from the desensitization curves. Currents were recorded in outside-out patch recordings (-60 mV) from cells transfected with GluA, GluA + ABHD6, GluA + TARP-γ-2, and GluA + TARP-γ-2 + ABHD6. The number of patches is given in parentheses.

^a^Significantly different from GluA.

^b^Significantly different from GluA + TARP γ-2.
