# Supplementary material for: α/β-Hydrolase domain-containing 6 (ABHD6) accelerates the desensitization and deactivation of TARP γ-2-containing AMPA receptors
Source: eLife. 2026 Aug 18;13:RP99623. doi: 10.7554/eLife.99623 (PMC13485305; doi:10.7554/eLife.99623)
Supplement: Supplementary file 17 [file elife-99623-supp6.docx]

Supplementary File 6 Summary of P values for comparison of τ _w, des_.

| **Subunit** | **GluA+ABHD6**  **vs. GluA** | **GluA+TARP γ-2 vs. GluA** | **GluA+TARP γ-2+ABHD6**  **vs. GluA+TARP γ-2** |
| --- | --- | --- | --- |
| GluA1i | P>0.9999(-) | P<0.0001(***) | P=0.0004(***) |
| GluA1o | P=0.2467(-) | P<0.0001(***) | P<0.0001(***) |
| GluA2(Q)i-R | P>0.9999(-) | P<0.0001(***) | P<0.0001(***) |
| GluA2(Q)o-R | P>0.9999(-) | P<0.0001(***) | P<0.0001(***) |
| GluA2(Q)i-G | P>0.9999(-) | P<0.0001(***) | P<0.0001(***) |
| GluA2(Q)o-G | P=>0.9999(-) | P<0.0001(***) | P<0.0001(***) |
| GluA2(Q)i-R-TARP γ-2 | P<0.001(***) | \ | \ |
| GluA1i-TARP γ-2 | P<0.001(***) | \ | \ |
| GluA2(Q)i-R-TARP γ-2  (+ 50 mV) | P<0.001(***) | \ | \ |
| GluA1i-TARP γ-2  (+ 50 mV) | P=0.013(*) | \ | \ |

P values for comparison of τ _w, des_ of GluAs when co-transfected with/without γ-2 or/and ABHD6. The data were analyzed using Prism 8 (GraphPad Software), and each P value was adjusted to account for multiple comparisons.
