# Supplementary material for: α/β-Hydrolase domain-containing 6 (ABHD6) accelerates the desensitization and deactivation of TARP γ-2-containing AMPA receptors
Source: eLife. 2026 Aug 18;13:RP99623. doi: 10.7554/eLife.99623 (PMC13485305; doi:10.7554/eLife.99623)
Supplement: Supplementary file 18 [file elife-99623-supp7.docx]

Supplementary File 7 Summary of τ _w, rec_ (ms) of GluAs when co-transfected with/without γ-2 or/and ABHD6.

| **Subunit** | **GluA** | **GluA**  **+ ABHD6** | **GluA**  **+ TARP γ-2** | **GluA**  **+ TARP γ-2**  **+ ABHD6** |
| --- | --- | --- | --- | --- |
| GluA1i | 223.90 ± 124.60(32/4) | 225.00 ± 201.39(29/4) | 80.66 ± 22.56(33/4)^a^ | 126.50 ± 58.83(30/4)^b^ |
| GluA1o | 182.60 ± 87.20(28/4) | 165.60 ± 39.86(25/4) | 100.70 ± 27.76(33/4)^a^ | 119.90 ± 41.56(26/4) |
| GluA2(Q)i-R | 77.17 ± 41.26(36/5) | 75.89 ± 32.54(38/5) | 56.98 ± 17.86(42/5)^a^ | 66.44 ± 33.63(27/5) |
| GluA2(Q)o-R | 67.60 ± 28.29(35/5) | 68.87 ± 36.06(33/5) | 54.05 ± 17.56(33/5) | 59.95 ± 26.61(33/5) |
| GluA2(Q)i-G | 52.29 ± 20.93(37/5) | 56.23 ± 37.72(29/5) | 40.85 ± 27.86(35/5)^a^ | 47.39 ± 26.72(31/5) |
| GluA2(Q)o-G | 50.48 ± 16.68(44/6) | 58.12 ± 33.49(39/6) | 50.39 ± 18.40(45/6) | 55.68 ± 29.88(35/6) |
| GluA2(Q)i-R-TARP γ-2 | 77.61± 20.99(63/9) | 82.66± 20.75(63/9) | \ | \ |
| GluA1i-TARP γ-2 | 93.89± 22.90(32/3) | 127.22± 11.96(31/3) | \ | \ |

Mean (± S.D.) values for constant of recovery from desensitization (τ _w, rec_) from the recovery ratio curves, which were calculated using nonlinear one-phase exponential decay fitting. Currents were recorded in outside-out patch recordings (-60 mV, 10 mM glutamate; 100 ms) when performing two-pulse stimulation from cells transfected with GluA, GluA + ABHD6, GluA + TARP γ-2, and GluA + TARP γ-2 + ABHD6. The number of patches is given in parentheses.

^a^Significantly different from GluA.

^b^Significantly different from GluA + TARP γ-2.
