# Supplementary material for: α/β-Hydrolase domain-containing 6 (ABHD6) accelerates the desensitization and deactivation of TARP γ-2-containing AMPA receptors
Source: eLife. 2026 Aug 18;13:RP99623. doi: 10.7554/eLife.99623 (PMC13485305; doi:10.7554/eLife.99623)
Supplement: Supplementary file 20 [file elife-99623-supp9.docx]

Supplementary File 9 Summary of GluA1i/GluA2(R)i-G receptors when co-transfected with/without γ-2 or/and ABHD6.

|  | **GluA1i/GluA2(R)i-G** | **GluA1i/GluA2(R)i-G + ABHD6** | **GluA1i/GluA2(R)i-G + TARP γ-2** | **GluA1i/GluA2(R)i-G + TARP γ-2 + ABHD6** |
| --- | --- | --- | --- | --- |
| Peak amplitude | 156.96 ± 140.07(31/5) | 65.75 ± 66.79(33/5) ^a^ | 492.72 ± 454.74(34/5)^a^ | 67.45 ± 141.71(32/5)^b^ |
| *τ* _w,_ _deact_ | 1.04 ± 0.46(31/5) | 1.00 ± 0.30(33/5) | 2.20 ± 0.98(34/5)^a^ | 1.59 ± 0.76(32/5)^b^ |
| τ _w, des_ | 3.85 ± 1.10(31/5) | 3.24 ± 0.76(33/5) | 7.77 ± 2.27(34/5)^a^ | 4.27 ± 1.53(32/5)^b^ |
| τ _rec_ | 129.72 ± 39.79(23/5) | 263.71 ± 180.45(19/5)^a^ | 83.11 ± 28.70(23/5)^a^ | 164.42 ± 86.02(17/5)^b^ |

Mean (± S.D.) values for peak amplitude, τ _w, des_ from the desensitization and *τ* _w,_ _deact_ from the deactivation curves, and τ _rec_ from the recovery ratio curves. Currents were recorded in outside-out patch recordings (- 60 mV, 10 mM glutamate; 100 ms / 1 ms / 100 two-pulse stimulation) without spermine in internal solution from cells transfected with GluA1i/GluA2(R)i-G, GluA1i/GluA2(R)i-G + ABHD6, GluA1i/GluA2(R)i-G + TARP γ-2, and GluA1i/GluA2(R)i-G + TARP γ-2 + ABHD6. The number of patches is given in parentheses.

^a^Significantly different from GluA1i/GluA2(R)i-G.

^b^Significantly different from GluA1i/GluA2(R)i-G + TARP γ-2.
