# Supplementary material for: α/β-Hydrolase domain-containing 6 (ABHD6) accelerates the desensitization and deactivation of TARP γ-2-containing AMPA receptors
Source: eLife. 2026 Aug 18;13:RP99623. doi: 10.7554/eLife.99623 (PMC13485305; doi:10.7554/eLife.99623)
Supplement: Peer review history

# Editor report: eLife Assessment

**DOI:** 10.7554/eLife.99623.3.sa0

**Authors:** Peng Cao

**Affiliations:** National Institute of Biological Sciences, Beijing China

**Keywords:** Compelling, Fundamental

This **fundamental** work demonstrates that ABHD6 regulates AMPAR gating kinetics in a TARP γ-2-dependent manner. The evidence in this study is **compelling**. This study will be of interest to readers in the field of synaptic transmission.

# Referee report: Reviewer #1 (Public review)

**DOI:** 10.7554/eLife.99623.3.sa1

Summary:

This research sheds light on the nuanced role of ABHD6 in regulating AMPARs, highlighting its interaction with TARP γ-2 as a critical factor in modulating receptor gating kinetics. It is crucial to understand that although ABHD6 alone does not alter AMPAR kinetics, its presence alongside TARP γ-2 accelerates AMPAR deactivation and desensitization, thereby affecting synaptic transmission dynamics.

Strengths:

Important findings in the research include:

- ABHD6 does not affect the gating kinetics of GluA1 and GluA2(Q) homomeric receptors independently.

- In the presence of TARP γ-2, ABHD6 accelerates deactivation and desensitization of these receptors, regardless of their splicing or editing isoforms.

- The effect is consistent for both homomeric GluA1 and GluA2(Q) receptors and heteromeric GluA1i/GluA2(R)i-G receptors.

- The recovery from desensitization of GluA1 with the flip splicing isoform is slowed by ABHD6 in the presence of TARP γ-2.

# Referee report: Reviewer #2 (Public review)

**DOI:** 10.7554/eLife.99623.3.sa2

Summary:

Cong et al. investigated the regulatory effects of ABHD6 on AMPARs. The authors performed adequate electrophysiology recordings to show the exact pattern of this regulation and covered major critical points.

Strengths:

The authors have performed high-quality ephys recordings and examined all potential regulatory aspects of ABHD6 on AMPARs. This is important to understand the AMPAR functions.

Weaknesses:

(1) The authors discussed CNIH-2 extensively from line 92-110 in the introduction, however, they did not perform related experiments. I suggest they move this part to the discussion where they also discussed the roles of CNIH.

(2) The authors need to report the "n" for all the experiments they have presented in this manuscript. How many cells were recorded in each condition? How many batches? This information has to be in all of the figure legends, but it is missing except Fig. 4.

(3) One question is what the physiological meanings of this regulatory effect are. The authors may consider adding some discussions.

(4) About statistics. The authors need to add more details and make sure their statistics sound. For example, they also need to check the equality of variances. In their Table EVs, where the P values are reported, the authors need to report which statistics they have used, one-way ANOVA, K-W test, or others, and the exact post-hoc test type for each comparison. For one-way ANOVA, report the F values simultaneously with the P values in all figure legends.

(5) Fig. 3J, the authors need to correct the label of the Y axis. It is shifted.

Comments on revised version.

In the revised manuscript, the authors have addressed all my concerns. The manuscript has been substantially strengthened by additional data and discussion.

# Author comment: Author response

**DOI:** 10.7554/eLife.99623.3.sa3

**Authors:** Rixu Cong, Huiran Li, Hong Yang, Jing Gu, Shanshan Wang, Qi Liu, Xiangyu Guan, Tangyunfei Su, Yulin Zheng, Dianchun Wang, Xinran Chen, Lei Yang, Yun Stone Shi, Mengping Wei, Chen Zhang

**Affiliations:** Capital Medical University Beijing China; Capital Medical University Beijing China; Capital Medical University Beijing China; Capital Medical University Beijing China; Peking University Beijing China; Capital Medical University Beijing China; Capital Medical University Beijing China; Capital Medical University Beijing China; Wenzhou Medical University Wenzhou China; Capital Medical University Beijing China; Capital Medical University Beijing China; Capital Medical University Beijing China; Nanjing University Nanjing China; Capital Medical University Beijing China; Capital Medical University Beijing China

The following is the authors’ response to the original reviews.

**Public Reviews:**

**Reviewer #1 (Public Review):**

Summary:

This research sheds light on the nuanced role of ABHD6 in the regulation of AMPARs, highlighting its interaction with TARP γ-2 as a critical factor in modulating receptor-gating kinetics. It is crucial to understand that while ABHD6 alone does not alter AMPAR kinetics, its presence alongside TARP γ-2 leads to accelerated deactivation and desensitization of AMPARs, impacting synaptic transmission dynamics.

Strengths:

Important findings in the research include:

ABHD6 does not affect the gating kinetics of GluA1 and GluA2(Q) homomeric receptors independently.

In the presence of TARP γ-2, ABHD6 accelerates deactivation and desensitization of these receptors, regardless of their splicing or editing isoforms.

The effect is consistent for both homomeric GluA1 and GluA2(Q) receptors and heteromeric GluA1i/GluA2(R)i-G receptors.

The recovery from desensitization of GluA1 with the flip splicing isoform is slowed by ABHD6 in the presence of TARP γ-2.

We are grateful for the reviewer's positive comments. It is really exciting to have one’s comments like “This research sheds light on the nuanced role of ABHD6 in the regulation of AMPARs”.

Weaknesses:

However, the study focuses on specific receptor subunits and isoforms, which may not fully represent the diversity of AMPAR compositions found in vivo (e.g. though the authors have claimed that TARP γ-2 failed to increase GluA3-induced currents significantly, the effect on GluA4 or the explanation was missing). Further research is needed to explore the implications of these findings in more complex neuronal environments.

Thank the reviewer for raising this point. To investigate whether ABHD6 is involved in the kinetic regulation of neurons, we recorded glutamate-induced currents at –70 mV using ABHD6 knockout neurons. We found that ABHD6 knockout neurons exhibited significantly slower deactivation and desensitization kinetics (Fig. 6, Table. EV7.1, EV7.2). Regarding the diversity of AMPAR subunit compositions, we obtained consistent results for GluA4, which is expressed at higher levels in the cerebellum and brainstem (Fig. 7, EV7, Table EV8.1, EV8.2). Specifically, we observed that ABHD6 accelerates the deactivation and desensitization of homomeric GluA4–TARP γ-2 complexes.

**Reviewer #2 (Public Review):**

Summary:

Cong et al. investigated the regulatory effects of ABHD6 on AMPARs. The authors performed adequate electrophysiology recordings to show the exact pattern of this regulation and covered major critical points.

Strengths:

The authors have performed high-quality ephys recordings and examined all potential regulatory aspects of ABHD6 on AMPARs. This is important to understand the AMPAR functions.

We greatly appreciate the reviewer’s positive comment on our manuscript and recognition of our quality ephys recordings.

Weaknesses:

(1) The authors discussed CNIH-2 extensively from line 92-110 in the introduction, however, they did not perform related experiments. I suggest they move this part to the discussion where they also discussed the roles of CNIH.

We thank the reviewer for the suggestions. Accordingly, we have moved the discussion of CNIH‑2 to the Discussion section (lines 355–372) of the revised manuscript: “Other key modulators include cornichon family AMPA receptor auxiliary proteins (CNIH-2/3) and GSG1L, which generally slow receptor kinetics in heterologous expression systems (Kato et al., 2010; Schwenk et al., 2012), although their effects in neurons can be context-dependent (Gu et al., 2016; Mao et al., 2017). Additional diversity arises from synapse-enriched proteins such as SynDIG4 and CKAMP44, which exert complex and sometimes opposing effects on different kinetic parameters (Matt et al., 2018; Khodosevich et al., 2014). This diversity comes from the known co-assembly of AMPA receptor subunits (the pore-forming GluA subunit) with three classes of auxiliary proteins—collectively comprising 21 components, most of which are secretory or transmembrane proteins. Importantly, multiple auxiliary subunits (e.g., TARP γ-8 and CNIH-2) can co-assemble within a single AMPAR complex, and their combined presence modulates functional outcomes in ways not predicted by individual subunits alone, underscoring a combinatorial regulatory logic (Shi et al., 2010; Yu et al., 2021; Herring et al., 2013). Given that native synaptic AMPARs predominantly exist as GluA2-containing hetero-oligomers (e.g., GluA1/2, GluA2/3), although homo-oligomers have also structurally validated, understanding how novel auxiliary proteins such as ABHD6 integrate into this complex framework becomes paramount (Lu et al., 2009; Wenthold et al., 1996; Zhao et al., 2016; Malinow and Malenka, 2002).”

(2) The authors need to report the "n" for all the experiments they have presented in this manuscript. How many cells were recorded in each condition? How many batches? This information has to be in all of the figure legends, but it is missing except Fig. 4.

We appreciate the reviewer for pointing out these weaknesses, we added the cell number and corresponding batches in every figure and table in the revised manuscript.

(3) One question is what the physiological meanings of this regulatory effect are. The authors may consider adding some discussions.

We thank the reviewer for the suggestions. In the revised manuscript, we have included a discussion on the physiological implications of this regulatory effect in lines 386–412, as follows: “Although there is no direct evidence indicating that ABHD6 and TARP γ-2 bind to each other, both are known to associate with AMPA receptors, suggesting the possibility of indirect or regulatory interactions. For example, their relationship could be transient, condition-dependent, or mediated through mechanisms such as conformational changes or steric hindrance (Gill et al., 2011b; Sumioka, 2013; Wei et al., 2017). Studies have reported that scaffold proteins participate in the binding, anchoring, maintenance, and removal of AMPA receptors, either through direct interaction with receptors or through indirect binding via auxiliary subunits (Danielson et al., 2014). Additionally, we extended the same experimental approach to AMPA receptors containing the GluA1 flip subtype together with TARP γ-8. Our results demonstrate that this ABHD6-dependent regulatory mechanism also applies to other TARP family members, including TARP γ-8 (Figure 7, EV7, Table. EV9.1, EV9.2). Our findings indicate that ABHD6 plays a critical negative regulatory role on AMPA receptor function. It suppresses synaptic current amplitude and accelerates the deactivation and desensitization kinetics in a TARP γ-2-dependent manner. By shortening synaptic response duration and reducing total charge transfer, ABHD6 may thereby restrain neuronal excitability and narrow the temporal window for synaptic integration. Loss of ABHD6 function—as observed in our knockout neurons, which exhibit slowed kinetics—could promote excitatory hyperactivity. Thus, as a key “molecular brake” on synaptic excitability, dysregulation of ABHD6 may directly contribute to the pathogenesis of neurological disorders. Insufficient braking function may lead to excessive synaptic transmission, strongly correlating with hyperexcitability conditions such as epilepsy. Conversely, overly potent braking might result in synaptic dysfunction, potentially contributing to early synaptic impairment in cognitive disorders like Alzheimer’s disease. Overall, our research highlights ABHD6 as a promising target for novel therapeutic strategies in neurological disorders and provides a solid theoretical foundation for further investigation in this field.”

(4) About statistics. The authors need to add more details and make sure their statistics sound. For example, they also need to check the equality of variances. In their Table EVs, where the P values are reported, the authors need to report which statistics they have used, one-way ANOVA, K-W test, or others, and the exact post-hoc test type for each comparison. For one-way ANOVA, report the F values simultaneously with the P values in all figure legends.

We appreciate your thoughtful advice. Accordingly, we have added the description of statistical strategy in the revised manuscript in line 530-536: “Data were first assessed for normality using the D’Agostino–Pearson test (n＜50) or the Kolmogorov-Smirnov test (n＞50), and for equality of variances using the Brown-Forsythe ANOVA test. Depending on the outcome of these tests, data were analyzed by parametric (one-way ANOVA) or non-parametric methods (Kruskal-Wallis test) followed by Tukey's Honest Significant Difference (HSD) test as a post hoc analysis to determine specific differences among groups. Correlation was evaluated with Pearson correlation analysis. Values of P < 0.05 were considered statistically significant.”

(5) Fig. 3J, the authors need to correct the label of the Y axis. It is shifted

Thank the reviewer for raising this point, we have corrected the label of the Y axis of Fig. 3J in the revised manuscript.

**Recommendations for the authors:**

**Reviewer #1 (Recommendations For The Authors):**

The manuscript is well-structured and the findings are presented clearly. While the study addresses multiple isoforms, a more detailed explanation of the isoform-specific effects observed, e.g. the unique behavior of the GluA2(Q)i-G isoform in terms of deactivation, would be beneficial.

We appreciate the reviewer for pointing out these weaknesses. In response, we have added a discussion in the revised manuscript in line 330-345 that addresses RNA editing as a key regulatory mechanism of AMPAR function beyond subunit composition and splicing variants: “Beyond subunit composition and splicing variants, the function of AMPARs is also finely regulated by RNA editing. Q/R editing enables the conversion of neutral to positively charged residues in the ion-selective filter of the channel, causing impermeability to divalent cations such as Ca^2+^. This not only alters channel conductance and current but also contributes to neuronal dysfunction and excitotoxicity (Kawahara et al., 2004; Kwak and Kawahara, 2004). R/G editing markedly influences receptor desensitization and recovery kinetics, and may modulate interactions with auxiliary proteins, thereby playing a critical role in synaptic plasticity and development (Stern-Bach et al., 1998; Coombs et al., 2012; Wright and Vissel, 2012). The conversion from R to G weakens inter-dimer interactions within the binding domains, leading to structurally more flexible receptors (Lomeli et al., 1994). Furthermore, R/G editing exhibits strong developmental regulation and varies across brain regions and cell types (Geiger et al., 1995). Therefore, in this study, we systematically examined the effect of ABHD6 on different flip/flop splice variants and R/G editing subtypes. Our results demonstrate that ABHD6 also suppresses currents in HEK 293T cells expressing flop splice variants and R/G-edited receptors.”

The authors should consider discussing potential mechanisms underlying the interaction between ABHD6 and TARP γ-2 in greater depth. This could include hypotheses on how ABHD6 might be influencing TARP γ-2's modulation of AMPARs if applicable (though the authors have mentioned either the potential binding domain of ABHD6 to AMPARs or TARP γ-2 to AMPARs, the proposed direct interaction between ABHD6 and TARP γ-2 is unknown). It's also unclear whether the effect of ABHD6 is specific to TARP γ-2 or is general to other TARP family members.

We appreciate your suggestion and use affinity chromatography to examine the interaction between ABHD6 and TARP γ-2. Our investigation revealed no direct evidence of a physical binding between the two proteins. Accordingly, we have supplemented the discussion in the revised manuscript (lines 386–393) as follows: “Although there is no direct evidence indicating that ABHD6 and TARP γ-2 bind to each other, both are known to associate with AMPA receptors, suggesting the possibility of indirect or regulatory interactions. For example, their relationship could be transient, condition-dependent, or mediated through mechanisms such as conformational changes or steric hindrance (Gill et al., 2011b; Sumioka, 2013; Wei et al., 2017). Studies have reported that scaffold proteins participate in the binding, anchoring, maintenance, and removal of AMPA receptors, either through direct interaction with receptors or through indirect binding via auxiliary subunits (Danielson et al., 2014).”

Expanding the discussion to include the potential physiological and pathophysiological implications of ABHD6's modulatory effects on AMPAR kinetics would provide a broader context for the findings.

We thank the reviewer for the suggestions, in the revised manuscript we discussed the physiological meanings of this regulatory effect in line 386-412: “Although there is no direct evidence indicating that ABHD6 and TARP γ-2 bind to each other, both are known to associate with AMPA receptors, suggesting the possibility of indirect or regulatory interactions. For example, their relationship could be transient, condition-dependent, or mediated through mechanisms such as conformational changes or steric hindrance (Gill et al., 2011b; Sumioka, 2013; Wei et al., 2017). Studies have reported that scaffold proteins participate in the binding, anchoring, maintenance, and removal of AMPA receptors, either through direct interaction with receptors or through indirect binding via auxiliary subunits (Danielson et al., 2014). Additionally, we extended the same experimental approach to AMPA receptors containing the GluA1 flip subtype together with TARP γ-8. Our results demonstrate that this ABHD6-dependent regulatory mechanism also applies to other TARP family members, including TARP γ-8 (Figure 7, EV7, Table. EV9.1, EV9.2). Our findings indicate that ABHD6 plays a critical negative regulatory role on AMPA receptor function. It suppresses synaptic current amplitude and accelerates the deactivation and desensitization kinetics in a TARP γ-2-dependent manner. By shortening synaptic response duration and reducing total charge transfer, ABHD6 may thereby restrain neuronal excitability and narrow the temporal window for synaptic integration. Loss of ABHD6 function—as observed in our knockout neurons, which exhibit slowed kinetics—could promote excitatory hyperactivity. Thus, as a key “molecular brake” on synaptic excitability, dysregulation of ABHD6 may directly contribute to the pathogenesis of neurological disorders. Insufficient braking function may lead to excessive synaptic transmission, strongly correlating with hyperexcitability conditions such as epilepsy. Conversely, overly potent braking might result in synaptic dysfunction, potentially contributing to early synaptic impairment in cognitive disorders like Alzheimer’s disease. Overall, our research highlights ABHD6 as a promising target for novel therapeutic strategies in neurological disorders and provides a solid theoretical foundation for further investigation in this field.”.

Some typos:

p7L144, might miss a word 'of' after 'properties';

Thanks for your careful advice, we have corrected “the channel properties TARP γ-2-containing AMPA receptors” to “the channel properties of TARP γ-2-containing AMPA receptors” in the revised manuscript.

p9L178, remove '.';

Thanks for your careful advice, we have corrected the subheading “ABHD6 accelerated the deactivation of homomeric AMPAR-TARP γ-2 complexes.” to “ABHD6 accelerated the deactivation of homomeric AMPAR-TARP γ-2 complexes” in the revised manuscript.

p9L195, might be 'deact' instead of 'deac';

Thanks for your careful advice, we have corrected “τ_w, deac_” to “τ _w, deact_ " in the revised manuscript.

p12L276, might be a missing 'ABDH6' after 'whether'.

Thanks for your advice, we have added “ABHD6” after “whether” in the revised manuscript.

**Reviewer #2 (Recommendations For The Authors):**

(1) Line, 366, grammar mistake. The author used the expression "In this study, we systematically studies", which should be “study" instead of :”studies"

Thanks for your advice, we have corrected “studies” to “study” in the revised manuscript.

(2) Line 370, the author used the expression "However, previous studies also found poorly expressed but significant population of GluA1 homomeric receptors in the hippocampus". It looks like "poorly expressed" is somewhat contradictory to "significant". I suggest the authors revise this sentence.

Thanks for your advice, we have deleted the statement in the revised manuscript.

(3) Line 407-409. The authors stated, "The flip and flop isoforms were cloned into an IRES-GFP expression vector using polymerase chain reaction (PCR). ...editing variants were generated using PCR". It is impossible to use PCR only to finish all cloning, especially with IRES-GFP. This must be done via restriction enzyme, or Gibson assembly, or another method. The author probably PCRed the isoforms and then put them into the vectors using other methods. The authors need to revise their statement and make it complete and clear.

We thank the reviewer for their suggestion. In response, we have added a description of the expression vector construction to the revised manuscript in line 431-437: “The flip and flop isoforms were cloned into an IRES-GFP expression vector using polymerase chain reaction (PCR). Q/R and R/G editing variants were generated by PCR-based cloning and FastCloning. GluA1 and TARP γ-2 were subcloned using EcoRI and SalI sites (Milstein et al., 2007), GluA2 and GluA3 were inserted with XhoI and SalI, and GluA4 was inserted with EcoRI and BamHI. All constructs were verified by restriction mapping and sequencing of PCR-amplified regions.”

(4) It would help if the authors could show some WB blots or PCR results or other evidence that their transfection was successful, in particular with these many plasmid combinations.

We thank the reviewer for raising this point. In response, we have included additional experiments in the revised manuscript in line 138-142: “Immunofluorescence assays and Western blot analysis were performed on cells co-transfected with GluA1, TARP γ-2, and ABHD6. These experiments were conducted to verify co-transfection efficiency and corresponding protein expression. Immunofluorescence results confirmed a high degree of co‑localization among GluA1, TARP γ-2, and ABHD6 (Fig. EV1).”
